# Supplementary material for: 2015 versus 2021: Self-Reported Preparedness to Prescribe Antibiotics Prudently among Final Year Medical Students in Sweden
Source: Antibiotics (Basel). 2024 Mar 27;13(4):303. doi: 10.3390/antibiotics13040303 (PMC11047482; doi:10.3390/antibiotics13040303)
Supplement: Supplementary file 1 [file antibiotics-13-00303-s001.zip › ANTIBIOTICS Student-PREPARE followup Sweden SUPPLEMENTARY MATERIALS 240305.pdf]

2015 versus 2021: self-reported preparedness to prescribe antibiotics prudently among final year medical students in Sweden

Supplementary materials

Figure S1: Response rate at each medical school, 2015 and 2021

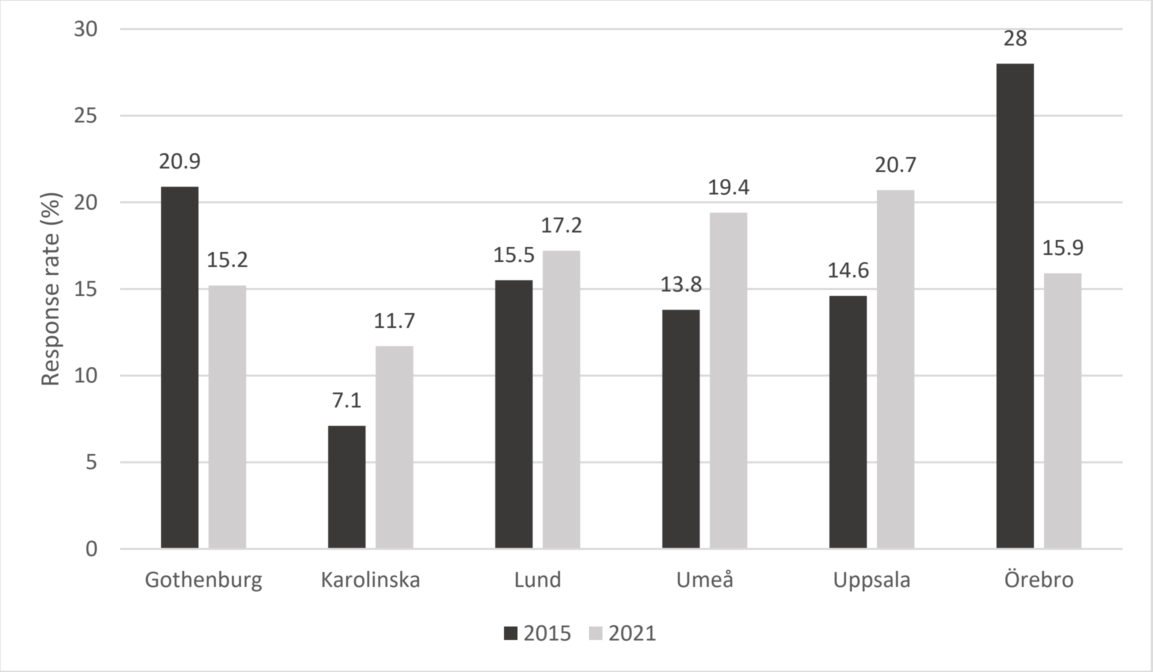

Table S1: Differences in global preparedness scores between medical schools, 2015 and 2021.

Medical school	*Global preparedness score 2015 (%)*	*Global preparedness score 2021 (%)*	*P-value*
Gothenburg University	80.5	78	0.0616
Karolinska Institutet	83.3	79.8	**0.0042**
Lund University	82.2	79.1	0.0666
Umeå University	82.3	69.3	**<0.001**
Uppsala University	85.9	82.5	0.0526
Örebro University	84.9	73.4	**<0.001**
Average	83.2	77	**<0.001**

Table S2. Absolute percentage change in curriculum preparedness score in 2021 versus 2015 per medical school.

Topic	Absolute change[1] in preparedness score per topic (%)					
	Gothenburg	Karolinska	Lund	Umeå	Uppsala	Örebro
To recognize the clinical signs of infection	0.0	0.0	-0.3	0.8	0.0	0.0
To assess the clinical severity of infection (e.g. using criteria, such as septic shock criteria)	5.9	-0.7	-6.5	-7.5	-3.9	-11.1
To use point-of-care tests (e.g. urine dipstick, rapid diagnostic tests for streptococcal pharyngitis)	-0.9	3.9	4.4	-14.8	-10.3	-7.7
To interpret biochemical markers of inflammation (e.g. CRP)	-0.3	-0.5	2.9	4.3	-1.7	0.3
To decide when it is important to take microbiological samples before starting antibiotic therapy	-6.9	0.9	1.0	-5.9	-0.3	0.3
To interpret basic microbiological investigations (e.g. blood cultures, antibiotic susceptibility reporting)	-6.8	6.0	1.0	-1.6	-5.3	-2.1
To identify clinical situations when not to prescribe an antibiotic	-2.6	-2.7	-14.3	-8.9	-7.0	-30.2
To differentiate between bacterial colonization and infection (e.g. asymptomatic bacteriuria)	-4.9	-3.3	2.1	-6.2	-2.0	-19.1
To differentiate between bacterial and viral upper respiratory tract infections	-8.9	-9.8	-6.0	-19.1	-2.6	-25.0
To select initial empirical therapy based on the most likely pathogen(s) and antibiotic resistance patterns, without using guidelines	-12.9	-10.0	-8.7	-26.6	2.6	-15.7
To decide the urgency of antibiotic administration in different situations (e.g. <1 hr for severe sepsis, non-urgent chronic bone infections)	9.4	-5.9	-17.7	-14.5	-0.9	4.1
To prescribe antibiotic therapy according to national/local guidelines	3.6	-4.8	-8.1	-11.6	-4.2	-7.4
To assess antibiotic allergies (e.g. differentiating between anaphylaxis and hypersensitivity)	25.5	-3.2	-7.4	-15.3	-13.9	-18.5
To identify indications for combination antibiotic therapy	-5.4	-20.2	-8.2	-22.0	-8.0	-25.8
To decide the shortest possible adequate duration of antibiotic therapy for a specific infection	-24.9	0.4	-0.4	-31.7	-5.9	-31.7
To prescribe using principles of surgical antibiotic prophylaxis	-25.8	0.3	-4.6	-22.0	-14.5	6.4
To review the need to continue or change antibiotic therapy after 48-72 hours, based on clinical evolution and laboratory results	-3.8	-0.5	11.9	-13.4	-25.6	1.6
To assess clinical outcomes and possible reasons for failure of antibiotic treatment	-1.5	-4.4	-3.4	-12.3	-1.2	-4.2
To decide when to switch from intravenous (IV) to oral antibiotic therapy	-4.2	-13.4	-8.4	-22.0	-6.8	-18.1

To measure/audit antibiotic use in a clinical setting, and to interpret the results of such studies	-7.8	-17.9	-7.9	-30.6	-11.2	-25.2
To work within the multi-disciplinary team in managing antibiotic use in hospitals	1.3	1.3	5.4	-2.0	-6.7	-17.2
To discuss antibiotic use with patients who are asking for antibiotics, when I feel they are not necessary	-4.5	3.9	2.1	-6.7	1.1	-7.7
To communicate with senior doctors in situations where I feel antibiotics are not necessary, but I feel I am being inappropriately pressured into prescribing antibotics by senior doctors	-9.2	-26.5	-0.4	-12.8	-12.6	-48.4
To use knowledge of the common mechanisms of antibiotic resistance in pathogens	-1.4	-4.5	5.9	-18.8	-0.9	-30.6
To use knowledge of the epidemiology of bacterial resistance, including local/regional variations	-10.3	-9.5	-13.1	-40.6	-3.5	-24.0
To practice effective infection control and hygiene (to prevent spread of bacteria)	-6.6	-2.6	-3.1	-0.3	-1.7	-5.6
To use knowledge of the negative consequences of antibiotic use (bacterial resistance, toxic/adverse effects, cost, Clostridium difficile infections)	-2.3	2.4	-3.1	-8.6	-1.7	3.1

[1] A negative result indicates a lower score in 2021 than in 2015.
