# Supplementary material for: 2015 versus 2021: Self-Reported Preparedness to Prescribe Antibiotics Prudently among Final Year Medical Students in Sweden
Source: Antibiotics (Basel). 2024 Mar 27;13(4):303. doi: 10.3390/antibiotics13040303 (PMC11047482; doi:10.3390/antibiotics13040303)
Supplement: Supplementary file 1 [file antibiotics-13-00303-s001.zip › Student-PREPARE 2021 survey.pdf]

1. In which age category are you?

- ◯ 20-25
- ◯ 26-30
- ◯ 31-35
- ◯ ≥36

2. What is your sex?

- ◯ Female
- ◯ Male
- ◯ Do not want to answer

3. Which medical school are you studying at?

- ◯ Karolinska Institutet
- ◯ Uppsala University
- ◯ Umeå University
- ◯ Lund University
- ◯ Gothenburg University
- ◯ Örebro University
- ◯ Linköping University

4. Which semester are you currently studying in medical school?

- ◯ 10
- ◯ 11
- ◯ Other (please specify)

5. During which semester do you have your infectious diseases course and clinical rotation?

- ☐ 5
- ☐ 6
- ☐ 7
- ☐ 8
- ☐ 9
- ☐ 10
- ☐ 11
- ☐ Other (please specify)

* 6. Diagnosis of infection

I feel...

	No teaching was provided	1 (Not at all prepared)	2	3	4 (Sufficiently prepared)	5	6	7 (Very well prepared)	I am unsure how I feel	I do not understand the question
To recognise the clinical signs of infection	○	○	○	○	○	○	○	○	○	○
To assess the clinical severity of infection (e.g using criteria, such as the septic shock criteria)	○	○	○	○	○	○	○	○	○	○
To use point- of-care tests (e.g. urine dipstick, rapid diagnostic tests for streptococcal pharyngitis)	○	○	○	○	○	○	○	○	○	○
To interpret biochemical markers of inflammation (e.g. CRP)	○	○	○	○	○	○	○	○	○	○
To decide when it is important to take microbiological samples before starting antibiotic therapy	○	○	○	○	○	○	○	○	○	○
To interpret basic microbiological investigations (e.g. blood cultures, antibiotic susceptibility reporting)	○	○	○	○	○	○	○	○	○	○

* 7. Indications for no antibiotic treatment

I feel...

	No teaching was provided	1 (Not at all prepared)	2	3	4 (Sufficiently prepared)	5	6	7 (Very well prepared)	I am unsure how I feel	I do not understand the question
To identify clinical situations when not to prescribe an antibiotic										
To differentiate between bacterial colonisation and infection (e.g. asymptomatic bacteriuria)										
To differentiate between bacterial and viral upper respiratory tract infections										

* 8. Initial anibiotic therapy

I feel...

	No teaching was provided	1 (Not at all prepared)	2	3	4 (Sufficiently prepared)	5	6	7 (Very well prepared)	I am unsure how I feel	I do not understand the question
To select initial empirical therapy based on the most likely pathogen(s) and antibiotic resistance patterns, without using guidelines										
To decide the urgency of antibiotic administration in different situations (e.g <1 hr for severe sepsis, non- urgent for chronic bone infections)										
To prescribe antibiotic therapy according to national/local guidelines										
To assess antibiotic allergies (e.g. differentiating between anaphylaxis and hypersensitivity)										
To identify indications for combination antibiotic therapy										
To decide the shortest possible adequate duration of antibiotic therapy for a specific infection										
To prescribe using principles of surgical antibiotic prophylaxis										

* 9. Reassessment of antibiotic therapy

I feel...

	No teaching was provided	1 (Not at all prepared)	2	3	4 (Sufficiently prepared)	5	6	7 (Very well prepared)	I am unsure how I feel	I do not understand the question
To review the need to continue or change antibiotic therapy after 48-72 hours, based on clinical evolution and laboratory results	○	○	○	○	○	○	○	○	○	○
To assess clinical outcomes and possible reasons for failure of antibiotic treatment	○	○	○	○	○	○	○	○	○	○
To decide when to switch from intravenous (IV) to oral antibiotic therapy	○	○	○	○	○	○	○	○	○	○

* 10. Quality of care

I feel...

	No teaching was provided	1 (Not at all prepared)	2	3	4 (Sufficiently prepared)	5	6	7 (Very well prepared)	I am unsure how I feel	I do not understand the question
To measure/audit antibiotic use in a clinical setting, and to interpret the results of such studies	○	○	○	○	○	○	○	○	○	○
To work within the multi- disciplinary team in managing antibiotic use in hospitals	○	○	○	○	○	○	○	○	○	○

* 11. Communication skills

I feel...

	No teaching was provided	1 (Not at all prepared)	2	3	4 (Sufficiently prepared)	5	6	7 (Very well prepared)	I am unsure how I feel	I do not understand the question
To discuss antibiotic use with patients who are asking for antibiotics, when I feel they are not necessary	○	○	○	○	○	○	○	○	○	○
To communicate with senior doctors in situations where I feel antibiotics are not necessary, but I feel I am being inappropriately pressured into prescribing antibiotics by senior doctors	○	○	○	○	○	○	○	○	○	○

* 12. Antibiotic resistance

I feel...

	No teaching was provided	1 (Not at all prepared)	2	3	4 (Sufficiently prepared)	5	6	7 (Very well prepared)	I am unsure how I feel	I do not understand the question
To use knowledge of the common mechanisms of antibiotic resistance in pathogens	◯	◯	◯	◯	◯	◯	◯	◯	◯	◯
To use knowledge of the epidemiology of bacterial resistance, including local/regional variations	◯	◯	◯	◯	◯	◯	◯	◯	◯	◯
To practise effective Infection control and hygiene (to prevent spread of bacteria)	◯	◯	◯	◯	◯	◯	◯	◯	◯	◯
To use knowledge of the negative consequences of antibiotic use (bacterial resistance, toxic/adverse effects, cost, Clostridium difficile infections)	◯	◯	◯	◯	◯	◯	◯	◯	◯	◯

* 13. COVID 19
I feel...

	No teaching was provided	1 (Not at all prepared)	2	3	4 (Sufficiently prepared)	5	6	7 (Very well prepared)	I am unsure how I feel	I do not understand the question
To prescribe antibiotics for patients with severe covid-19	◯	◯	◯	◯	◯	◯	◯	◯	◯	◯

3. Teaching methods used for antibiotic education

14. Which of the following methods have been used at your medical school for teaching about prudent antibiotic use (the topics from the previous set of questions), and how useful were they?

	Teaching method was not used	Not at all useful	Neutral	Useful	Very useful	I am unsure	I do not understand the question
Lectures (with > 15 people)	○	○	○	○	○	○	○
Small group teaching (with <15 people)	○	○	○	○	○	○	○
Discussions of clinical cases and vignettes	○	○	○	○	○	○	○
Active learning assignments (e.g. article reading, group work, preparing an oral presentation)	○	○	○	○	○	○	○
E-learning Role play or communication skills sessions dealing with patients demanding antibiotic therapy	○	○	○	○	○	○	○
Infectious diseases clinical placement (i.e. clinical rotation or training in infectious diseases, involving patients)	○	○	○	○	○	○	○
Microbiology clinical placement	○	○	○	○	○	○	○
Peer or near-peer teaching (i.e. teaching led by other students, or recently qualified doctors)	○	○	○	○	○	○	○

Other used often (please specify)

15. Overall, do you feel you have received sufficient teaching at medical school in antibiotic use for your future practice as a junior doctor?

- ◯ Yes
- ◯ No, I feel I had enough teaching on general antibiotic treatment, but I need more on prudent antibiotic use
- ◯ No, I feel I need more education on both general antibiotic treatment and prudent antibiotic use
- ◯ I am unsure
- ◯ Other (please specify)

16. Has any of your teaching on antibiotic prescribing **changed format** due to the covid-19 pandemic e.g. from in-person to digital

- ◯ Yes
- ◯ No
- ◯ Unsure

17. Has any of your teaching on antibiotic prescribing **changed duration** due to the covid-19 pandemic?

- ◯ Yes, we have had more teaching
- ◯ Yes, we have had less teaching
- ◯ No
- ◯ Unsure

18. Do you believe that the **quality** of your teaching on antibiotic prescribing has been affected by the covid-19 pandemic?

- ◯ Much improved
- ◯ Improved
- ◯ No effect
- ◯ Worsened
- ◯ Much worsened
- ◯ Unsure

19. Do you feel overall that the covid-19 pandemic and its effects on your teaching has affected how prepared you feel to prescribe antibiotics prudently?

- ◯ Much improved
- ◯ Improved
- ◯ No effect
- ◯ Worsened
- ◯ Much worsened
- ◯ Unsure

20. Have you had teaching on prescribing antibiotics for patients with covid-19?

- ☐ Yes, by clinical supervisors
- ☐ Yes, at lectures, seminars or case-based discussions
- ☐ No
- ☐ Unsure
- ☐ Other (please specify)

21. Have any of your medical school examinations included questions on antibiotic treatment?

- ◯ Yes
- ◯ No
- ◯ Unsure
- ◯ Other (please specify)

22. How do you think teaching on antibiotic treatment and prudent antibiotic use can be improved?

4. Survey language and follow-up

23. How did you find the language used in this study?

- ◯ I could understand everything or almost everything
- ◯ I could understand most questions, it was alright for the survey to be in English
- ◯ Many questions were difficult to understand, I would have preferred the survey in my native language
- ◯ Most of the survey was difficult to understand, I would have preferred the survey in my native language
- ◯ Other (please specify)

Student-PREPARE 2021

Thank you for participating in Student-PREPARE 2021, we really appreciate the time you have taken to answer these questions, and we are very hopeful that this will help improve education on antibiotic use at medical schools in Sweden.
